# The chemistry of gut microbiome-derived lipopolysaccharides impacts on the occurrence of food allergy in the pediatric age

**DOI:** 10.3389/fmolb.2023.1266293

**Published:** 2023-10-13

**Authors:** Flaviana Di Lorenzo, Lorella Paparo, Laura Pisapia, Franca Oglio, Molly Dorothy Pither, Roberta Cirella, Rita Nocerino, Laura Carucci, Alba Silipo, Francesca de Filippis, Danilo Ercolini, Antonio Molinaro, Roberto Berni Canani

**Affiliations:** ^1^ Department of Chemical Sciences, University Federico II, Naples, Italy; ^2^ Task Force on Microbiome Studies, University Federico II, Naples, Italy; ^3^ Department of Translational Medical Science, University Federico II, Naples, Italy; ^4^ ImmunoNutritionLab at CEINGE Biotechnologies Research Center, University Federico II, Naples, Italy; ^5^ European Laboratory for Investigation of Food Induced Diseases, University Federico II, Naples, Italy; ^6^ Institute of Genetics and Biophysics, National Research Council, Naples, Italy; ^7^ Department of Agriculture, University Federico II, Naples, Italy; ^8^ Department of Chemistry, School of Science, Osaka University, Toyonaka, Osaka, Japan

**Keywords:** lipopolysaccharide (LPS), allergy, immune tolerance, Th2 response, Treg, gut microbiota, *Bacteroides* sp., lipid A

## Abstract

**Introduction:** Food allergy (FA) in children is a major health concern. A better definition of the pathogenesis of the disease could facilitate effective preventive and therapeutic measures. Gut microbiome alterations could modulate the occurrence of FA, although the mechanisms involved in this phenomenon are poorly characterized. Gut bacteria release signaling byproducts from their cell wall, such as lipopolysaccharides (LPSs), which can act locally and systemically, modulating the immune system function.

**Methods:** In the current study gut microbiome-derived LPS isolated from fecal samples of FA and healthy children was chemically characterized providing insights into the carbohydrate and lipid composition as well as into the LPS macromolecular nature. In addition, by means of a chemical/MALDI-TOF MS and MS/MS approach we elucidated the gut microbiome-derived lipid A mass spectral profile directly on fecal samples. Finally, we evaluated the pro-allergic and pro-tolerogenic potential of these fecal LPS and lipid A by harnessing peripheral blood mononuclear cells from healthy donors.

**Results:** By analyzing fecal samples, we have identified different gut microbiome-derived LPS chemical features comparing FA children and healthy controls. We also have provided evidence on a different immunoregulatory action elicited by LPS on peripheral blood mononuclear cells collected from healthy donors suggesting that LPS from healthy individuals could be able to protect against the occurrence of FA, while LPS from children affected by FA could promote the allergic response.

**Discussion:** Altogether these data highlight the relevance of gut microbiome-derived LPSs as potential biomarkers for FA and as a target of intervention to limit the disease burden.

## 1 Introduction

Pediatric food allergy (FA) is a serious health issue whose prevalence has increased over the past 30 years (Iweala and Nagler, 2019; Eisenstein, 2020). The occurrence of FA has been linked to alterations in the composition and function of gut microbiome (GM) (Iweala and Nagler, 2019; Eisenstein, 2020). Studies have suggested that specific GM signatures may have a potential role in promoting or inhibiting the development of FA (Feehley et al., 2019; Lee et al., 2020). In this frame, in our previous research activity within the MATFA (Microbiome As potential Target for innovative preventive and therapeutic strategies for Food Allergy) project (ClinicalTrials.gov Identifier: NCT04750980), we have identified a higher abundance of the Gram-positive *Ruminococcus gnavus* and a significant depletion of species belonging to the Gram-negative *Bacteroides* sp. in children with FA compared with healthy controls (De Filippis et al., 2021). These findings agreed with previous studies conducted at different time points in populations with different ethnicities, which further denoted the relevance of *Bacteroides* sp. in somehow protecting against the occurrence of FA (Ling et al., 2014; Tunpeng et al., 2021). The unveiled importance of *Bacteroides* sp. as potential protective microorganisms against FA seems controversial as Gram-negative bacteria are typically considered pathogens able to induce gut inflammation (Holmes et al., 2021). The inflammatory potential of Gram-negative bacteria is mainly associated with the major component of their outer membrane, the lipopolysaccharide (LPS) (Di Lorenzo et al., 2022). LPSs are glycoconjugates built up of three distinct moieties: i) a glycolipid portion called lipid A, ii) a relatively short oligosaccharide chain termed the core oligosaccharide (core OS), and iii) a highly variable polysaccharide named the O-antigen (Di Lorenzo et al., 2022). LPSs are potent microbial molecules able to boost the innate immunity in eukaryotes and to drive to sepsis in humans (Di Lorenzo et al., 2022). However, these effects are largely dependent on the LPS chemical structure (Di Lorenzo et al., 2022). In this light, we have recently shown that commensal *Bacteroides* strains express LPSs with uncommon structures exerting only weak or no inflammatory activity, and, in some cases, an anti-inflammatory action (Steimle et al., 2019; Di Lorenzo et al., 2020; Zhu et al., 2020; Pither et al., 2022a). Thus, *Bacteroides* sp., which comprise nearly 40% of GM in healthy children (Zafar and Saier, 2021), seem to possess LPSs unable to mount a proper inflammatory reaction, while possibly acting as tolerogenic agents (Steimle et al., 2019).

The MATFA project also aimed at evaluating the functional effect(s) of the GM in eliciting the allergic response by analyzing the fecal supernatants obtained from FA children and healthy age-matched controls (De Filippis et al., 2021). Once defined the microbiome taxonomic composition of fecal samples, we also have demonstrated that GM from FA children has a significant pro-allergic potential (De Filippis et al., 2021). This observation paved the way to further molecular studies searching for the microbial constituents likely responsible for this behavior. Here we focused on the LPS isolated from feces of healthy and FA children screened in the previous activities of the MATFA project. By using an analytical and organic chemistry approach, we identified distinctive lipid A and LPS structural features in healthy and FA children. In addition, we disclosed a different immunoregulatory action elicited by these diverse LPS and lipid A mixture on peripheral blood mononuclear cells (PBMCs), with LPS and lipid A derived from healthy individuals being able to protect against the occurrence of FA, while LPS and lipid A from FA children acting as promoters of the allergic response.

## 2 Methods

### 2.1 Preparation of fecal supernatants

The subject information sheet and the informed consent form were reviewed and approved by the Ethics Committee of the University of Naples Federico II (approval N. 2/14) and derive from our previous MATFA study (De Filippis et al., 2021). Fecal supernatants in fact were obtained from stool samples of CT (*n* = 10) and FA (*n* = 10) subjects, randomly selected from the dataset of study population of the MATFA (Microbiome As potential Target for innovative preventive and therapeutic strategies for Food Allergy) project (De Filippis et al., 2021). For all selected stool samples, we characterized gut microbiome features by shotgun metagenomics analyses. Fecal supernatants were prepared as previously described (De Filippis et al., 2021). Briefly, phosphate buffered saline (PBS) was added at equal volume to weight (2 g–2 mL) and was homogenized into a suspension, which underwent serial centrifugations at 1,700, 19,000 and 35,200 × *g* for 15 min each. The fecal supernatants were filtered with 0.22 µm filter and stored at −80°C until use. Then, to prepare samples for LPS and lipid A characterization, fecal supernatants were cyclically lyophilized (at least 3 lyophilization cycles were performed) by suspending each time dried samples in ∼10 mL of endotoxin-free water for tissue culture (ThermoFisher Scientific).

### 2.2 Extraction and purification of fecal LPS

300 mg of each dried fecal supernatant underwent an extensive enzymatic digestion with DNase (Roth) and RNase (Roth) (37°C, 16 h, under constant magnetic stirring), followed by a treatment with proteases (56°C, 16 h, under constant magnetic stirring) prior to the extraction step. Dialysis (cut-off 6–8 kDa) against distilled water was executed to eliminate contaminating proteins and nucleic acids. Once collected and lyophilized, each digested fecal supernatant underwent the hot phenol/water procedure (Westphal and Jann, 1965). Both water and phenol phases were inspected by means of SDS-PAGE followed by gel silver staining that revealed the occurrence of LPS only in the water phases. These phases were then ultracentrifuged (4°C, 208,000 × g, 24 h) and the precipitates containing LPS were further purified by means of a gel-filtration chromatography on a Sephacryl S-300 (GE Healthcare, 1.5 × 20 cm, eluent 50 mM NH_4_HCO_3_). The so-obtained fecal LPS were then washed 5 times with a mixture of CHCl_3_/CH_3_OH (1:2, vol/vol) and CHCl_3_/CH_3_OH/H_2_O (3:2:0.25, vol/vol) to remove phospholipids that were possibly present in the LPS preparations. After the removal of organic solvents and repeated cycles of lyophilization, each fecal LPS also underwent the “repurification” protocol previously described by Hirschfeld et al. (2000) to ensure the removal of any traces of lipoproteins/lipopeptides. A check via SDS-PAGE by using in parallel silver nitrate and Coomassie Brilliant Blue (Sigma-Aldrich) gel staining was executed to validate the purification step. In all SDS-PAGE analyses, the purified fecal LPS and the S-LPS from *Escherichia coli* 0127:B8 (Sigma-Aldrich) were suspended in endotoxin-free water for tissue culture (ThermoFisher Scientific) and prepared at the desired concentration (1 mg/mL), boiled for 10 min, and then 8 μL/well were loaded on a 13.5% SDS gel prepared with a 5% stacking gel and separated using a mini-PROTEAN electrophoresis instrument (Bio-Rad Laboratories).

### 2.3 Compositional analyses of fecal LPS

Monosaccharide content of purified fecal LPS was established by evaluation of the acetylated O-methyl glycoside (AMG) derivatives obtained by treating samples with HCl/CH_3_OH (1.25 M, 85°C, 16 h) followed by acetylation with acetic anhydride in pyridine (85°C, 25 min). The absolute configuration of each sugar residue was defined through the analysis of the acetylated *O*-octylglycoside (OGA) derivatives and comparison with authentic standards, as previously reported (Garcia-Vello et al., 2022a). The total fatty acid content was defined by treating an aliquot of each fecal LPS with 4M HCl (100°C, 4 h), followed by a treatment with 5M NaOH (100°C, 30 min). The pH was then adjusted to reach slight acidity followed by extraction of fatty acids in CHCl_3_, methylation with diazomethane, and analysis via Gas Chromatography Mass Spectrometry (GC-MS). Ester bound fatty acids were selectively released by base-catalyzed hydrolysis with aqueous 0.5 M NaOH/CH_3_OH (1:1, 85°C, 2 h), and then the product was acidified, extracted in CHCl_3_, methylated with diazomethane, and inspected via GC–MS. In addition, fecal LPS was also treated with 1.25M HCl/CH_3_OH (85°C, 16 h). The mixture was extracted three times with hexane. The hexane layer, which contained fatty acids as methyl esters derivatives, was then analyzed by GC–MS. All the above derivatives were analyzed on Agilent Technologies Gas Chromatograph 7820A equipped with a mass selective detector 5977B and an HP-5 capillary column (Agilent, Milan, Italy 30 m × 0.25 mm i. d., flow rate 1 mL/min, He as carrier gas). The temperature program used to analyze AMG and OGA was: 140°C for 3 min, then 140 → 240°C at 3°C/min. To analyze fatty acids content the following temperature program was used: 150°C for 5 min, 150°C–280°C at 3°C/min, and 280°C for 5 min.

### 2.4 MALDI-TOF MS analysis of fecal lipid A

To isolate the lipid A directly from feces, an aliquot of dried fecal supernatant (∼1 mg) was washed with 500 μL of CHCl_3_/CH_3_OH (1:2, vol/vol) and CHCl_3_/CH_3_OH/H_2_O (3:2:0.25, vol/vol) followed by several steps of centrifugation (1,200 × *g*, 30 min). Each washing mixture was first dried, weighed and checked via MALDI-TOF MS before being discarded. At this stage, fecal supernatants underwent a slightly modified El Hamidi et al. (2005) procedure which consisted in suspending samples in 200 μL of isobutyric acid/1 M ammonium hydroxide (5:3 vol/vol), followed by repeated cycles of quick (a few seconds) and alternating vortexing and ultrasonication steps. Then the reaction was conducted at 100°C for ∼1 h. Upon centrifugation (1,200 × g, 30 min) the supernatants were collected, diluted 1 to 4, lyophilized, and washed several times with methanol. Also in this case, all methanol washes were dried, weighed and checked via MALDI-TOF MS and then discarded. Following, a couple of washes with CHCl_3_/CH_3_OH/H_2_O (3:1.5:0.25, vol/vol) were performed. Once collected, the latter mixture was dried and checked by MALDI-TOF MS as previously described for small-scale extraction of lipid A from bacteria (El Hamidi et al., 2005). However, extremely low signal-to-noise ratio MS spectra were obtained, and in some cases a clear evidence of lipid A presence was not noticed. By contrast, MS spectra of better quality were clearly acquired when CHCl_3_/CH_3_OH (1:1, vol/vol) was used to suspend fecal lipid A mixture. All spectra were recorded in reflectron mode, both in negative- and positive-ion polarity, on an ABSCIEX TOF/TOF™ 5800 Applied Biosystems mass spectrometer equipped with an Nd:YAG laser (*λ* = 349 nm), with a 3 ns pulse width and a repetition rate of up to 1,000 Hz, and also equipped with delayed extraction technology. The matrix solution used in this study was 2,4,6-trihydroxyacetophenone (THAP) dissolved in CH_3_OH/0.1% trifluoroacetic acid/acetonitrile (7:2:1, vol/vol/vol) at a concentration of 75 mg/mL (Di Lorenzo et al., 2015a; Pither et al., 2022a), in place of 2,5-dihydroxybenzoic acid (DHB) in 0.1 M citric acid (El Hamidi et al., 2005). All experiments were in fact replicated using DHB, *α*-cyano-4-hydroxycinnamic acid, and 5-chloro-2-mercaptobenzothiazole as the matrixes, but several peaks were missing in most of the spectra, proving that these matrixes were not as useful as THAP in the analysis of fecal lipid A. Different ratios between the samples and matrix were also tested, with 1:1 providing best signal-to-noise ratio. Once deposited on the plate, the sample and the matrix solution were left to dry completely at room temperature before MALDI-TOF MS measurements. Lipid A from commercially available *E. coli* 0127:B8 LPS (Sigma-Aldrich), obtained by means of a home-made mild acid hydrolysis, was used as a standard. For MS experiments each spectrum was a result of the accumulation of 3,000 laser shots, whereas 5,000–7,000 shots were summed for the MS/MS spectra. Spots were randomly but evenly sampled for the range *m/z* 1,200–2,200. Spectra were calibrated and processed under computer control by using Data explorer software. All experiments were performed in technical triplicate and repeated on three different days with newly extracted fecal lipid A.

### 2.5 Blood sampling and isolation of peripheral blood mononuclear cells

Peripheral blood samples (8 mL) were obtained from three otherwise healthy children (Caucasian male, age range 48–61 months with negative clinical history for any allergic conditions and not at risk for atopic disorders), referred to the Department of Translational Medical Science at the University of Naples “Federico II” because of minimal surgical procedures. PBMCs were isolated by Ficoll density gradient centrifugation (Ficoll-Histopaque −1,077, Sigma, St. Louis, Missouri, United States). Briefly, cells were stratified on 3 mL of Ficoll and centrifuged 15 min at 1,000 × *g* at room temperature. After centrifugation, the opaque interface containing mononuclear cells was carefully aspirated with a Pasteur pipette and cells were washed with 10 mL of PBS and centrifuged 10 min at 500 × g at room temperature. After centrifugation, the upper layer was discarded and PBMCs were collected. Cells were cultured in duplicates in 96-well plates in 200 µL culture medium (RPMI 1640, Gibco) containing 10% FBS (Gibco), 1% non-essential amino acids (Gibco), 1% sodium pyruvate (Gibco), and 1% penicillin/streptomycin (Gibco).

### 2.6 PBMCs stimulation protocol and Th2, IL-10 and IL-12 cytokines determination

PBMCs (2 × 10^5^ cells/well) were stimulated with 5, 50, 100 μg/mL of fecal lipid A, LPS or supernatant for 6, 18, and 24 h in time-course and dose-response experiments. Cells with only medium were used as negative control. For subsequent experiments, we used 100 μg/mL for 24 h as the best dose and time. After incubation period, cells were harvested for flow cytometry and culture supernatants were collected to assess the Th2 cytokines (IL-4 and IL-13), IL-10 and IL-12 production. Concentration of these cytokines in supernatants of cells treated with lipid A, LPS or fecal was measured using the IL-4, IL-13, IL-10, and IL-12 human ELISA kit from Elabscience (Elabscience, Houston, Texas). The minimum detection concentration was 31.25 pg/mL for IL-4, 15.6 pg/mL for IL-13 and IL-12, and 1.6 pg/mL for IL-10. ELISAs were conducted according to the manufacturer’s recommendations.

### 2.7 Treg population and non-classical monocytes analyses by flow cytometry

Tregs were identified as CD4^+^/CD25^+^/FoxP3^+^ positive cells and non-classical monocytes were identified as CD14^+^/CD16^++^ positive cells by flow cytometry analysis. The staining for Tregs analyses was performed using Tregs detection human kit (Miltenyi Biotech, Bergisch Gladbach, Germany). CD14^+^ and CD16^++^ (Becton-Dickinson, Franklin Lakes, New Jersey, United States) was used as cell surface markers for the staining for non-classical monocyte. The results were analyzed by BD FACSCanto™ II flow cytometer and DIVA software (Becton-Dickinson). A total of 100,000 events were acquired for analysis after gating of lymphocytes based on the FSC/SSC dot plot.

### 2.8 Statistical analysis

The Kolmogorov-Smirnov test was used to determine whether variables were normally distributed. Descriptive statistics are reported as means and standard deviations for continuous variables. Differences among three groups were compared by one-way ANOVA test, followed by Tukey post-hoc test. Pearson’s correlation coefficient “r” was used to evaluate the correlation between continuous variables. Differences were considered statistically significant at *p* < 0.05. All data were collected in a dedicated database and analyzed by a using GraphPad Prism 7.

## 3 Results

### 3.1 Structural features of GM-derived LPS are different in FA children vs*.* healthy controls

From the subjects population analyzed in the previous MATFA study (De Filippis et al., 2021), we randomly selected stool samples of 10 children with FA and 10 healthy controls (CT) already evaluated for microbiome structure and functions (Supplementary Table S1). The samples were processed as previously described to obtain fecal supernatants (Marchesi et al., 2007; De Filippis et al., 2021). Supernatants then underwent a modified enzyme-hot phenol/water extraction procedure followed by several steps of purification and “re-purification” to exclude the co-presence of immunostimulatory contaminants, such as lipoproteins, lipopeptides and phospholipids, as previously described (Manthey and Vogel, 1994; Hirschfeld et al., 2000; Pither et al., 2023). The yield of LPS extracted from each fecal supernatant is reported in Supplementary Table S2. Surprisingly, we observed that the amount of LPS in the stool samples from FA patients was remarkably lower if compared with what observed in stool samples from CT.

Purified fecal LPSs from 6 FA (FA6, 11, 21, 22, 26, 28) and 6^−ΔΔCT^ (CT3, 10, 12, 20, 22, 25) children, chosen among those that resulted in the highest yield, were then checked via SDS-PAGE after silver nitrate gel staining (Supplementary Figures S1A, B). This analysis clearly highlighted that all CT children displayed a similar LPS profile, as proven by the polydisperse banding pattern (ladder-like pattern) due to the occurrence of LPS molecules with a different number of repeating units in the O-antigen moiety. By contrast, a rather diverse electrophoretic profile was observed for FA children derived LPS, with the presence of both LPS (Lanes 1 and 5, Supplementary Figure S1B) and LOS (Lanes 2–4 and 6, Supplementary Figure S1B), i.e., LPS devoid of the O-antigen repeating unit. No contaminating proteins/lipoproteins in the isolated fecal LPS extracts were detected (Supplementary Figures S1C, D).

**FIGURE 1:**
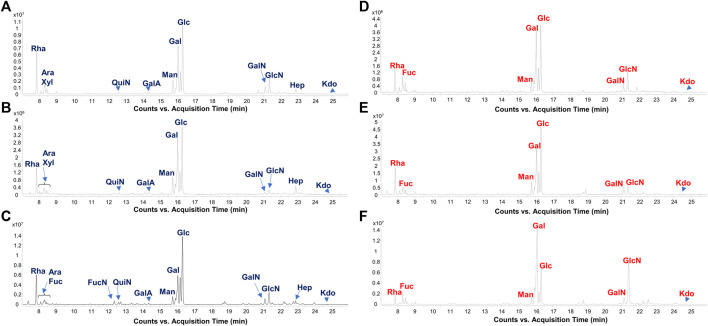
Gut microbiome-derived LPS present distinct carbohydrate profiles in CT vs. FA children. Zoom of GC-MS chromatogram profiles recorded after methanolysis followed by acetylation of an aliquot of the isolated LPS from FA [**(A–C)**, FA6, FA26, and FA21, respectively] and CT stool samples [**(D–F)**, CT3, CT12, and CT22, respectively] chosen as representatives among all twenty samples analyzed in this study. By this approach monosaccharides are detected as acetylated methyl glycosides. The chromatograms show the presence of Kdo in all samples, whereas heptoses (Hep) were only found in stool collected from FA patients. Abbreviations: Rha: rhamnose; Fuc: fucose; QuiN: quinovosamine; FucN: fucosamine; GalA: galacturonic acid; Man: mannose; Gal: galactose; Glc: glucose; GalN: galactosamine; GlcN: glucosamine; Hep: heptose.

The analysis of LPS carbohydrate and fatty acid content could provide clues on the LPS itself and about its source. Therefore, monosaccharides composing fecal LPS extracts were inspected and reported in Supplementary Table S3 (see also Figure 1). Remarkably, no heptose residues were detected in all fecal LPS extracts from the stool samples of the CT children, which were instead clearly observed in most of the LPS extracts collected by the stool samples of FA patients. These results are in agreement with the high abundance of *Bacteroides*-derived LPS in CT fecal samples (see below) as the absence of heptoses in the LPS of *Bacteroides* species is considered a hallmark of these bacteria (Di Lorenzo et al., 2019). By contrast, the presence of heptoses in FA fecal LPS suggested the presence of LPS from Gamma-proteobacteria, and possibly from the Enterobacteriaceae family whose blooming in the human gut typically leads to the instauration and persistence of an inflammatory state (Di Lorenzo et al., 2019; Di Lorenzo et al., 2022). Likewise, fatty acid compositional analyses disclosed the presence of the typical pattern of *Bacteroides* LPS in CT fecal LPS (Weintraub et al., 1989; Vatanen et al., 2016; Jacobson et al., 2018; Di Lorenzo et al., 2020; Pither et al., 2022b) whereas 12:0, 14:0, 14:0(3-OH) fatty acids were also identified in some FA fecal LPS, which might be considered instead a signature of Gamma-proteobacteria (Di Lorenzo et al., 2019) (Supplementary Figure S2).

### 3.2 GM-derived lipid A mass spectral profiles are diverse between FA children and healthy controls

**FIGURE 2:**
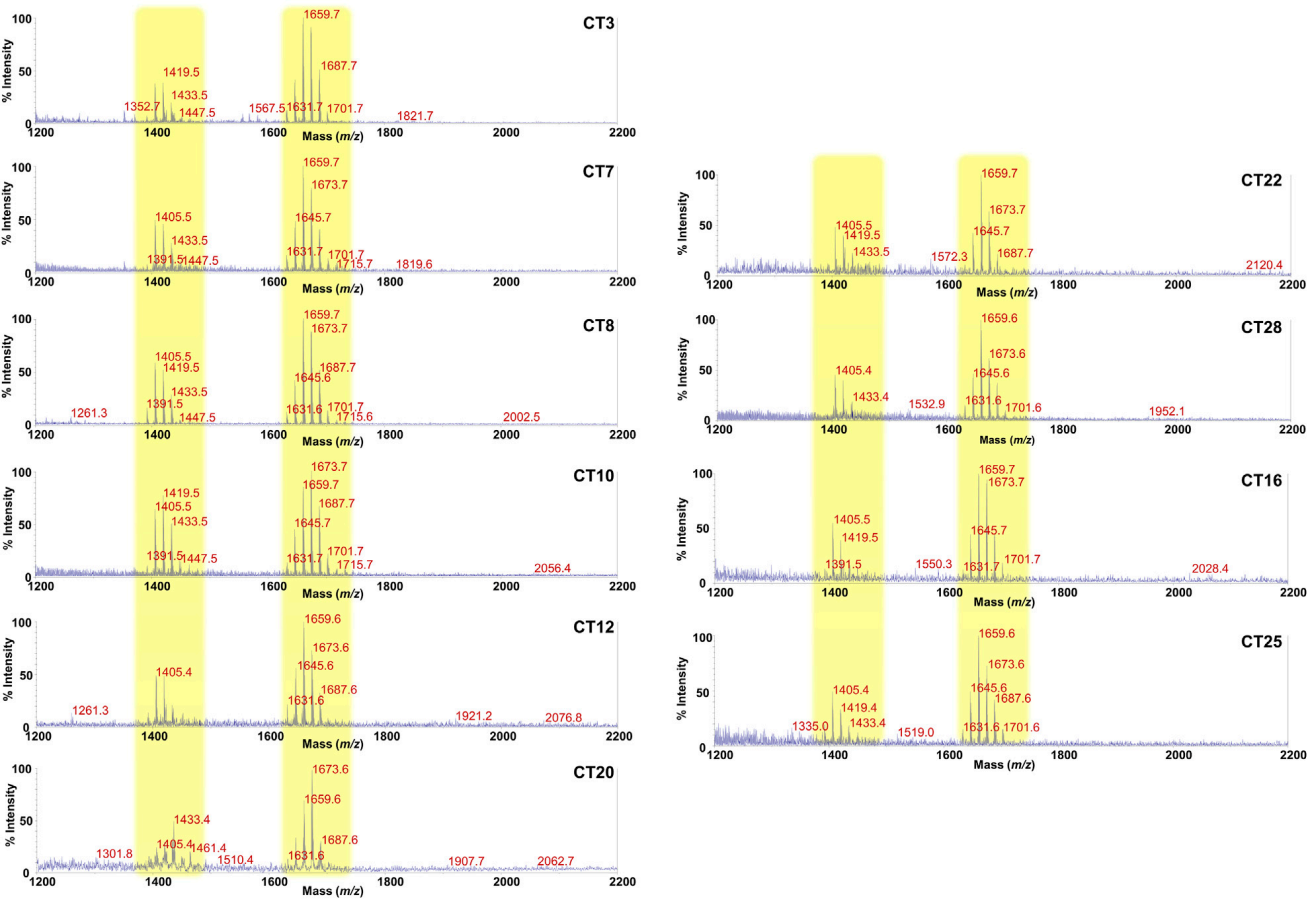
MALDI-TOF mass spectral profile of lipid A mixture extracted from fecal supernatants of healthy (CT) children. Reflectron MALDI-TOF MS spectra were recorded in negative ion polarity using THAP as the matrix. Yellow boxes highlight the clusters of peaks attributed to *mono*-phosphorylated tetra- (around *m/z* 1,419) and penta-acylated (around *m/z* 1,659) lipid A species from *Bacteroides* sp. Spectra were labeled “CT” followed by a number where CT indicates a healthy control child while the number is unique for each patient and is used to trace the patient information in the collection of samples of the MATFA project.

**FIGURE 3:**
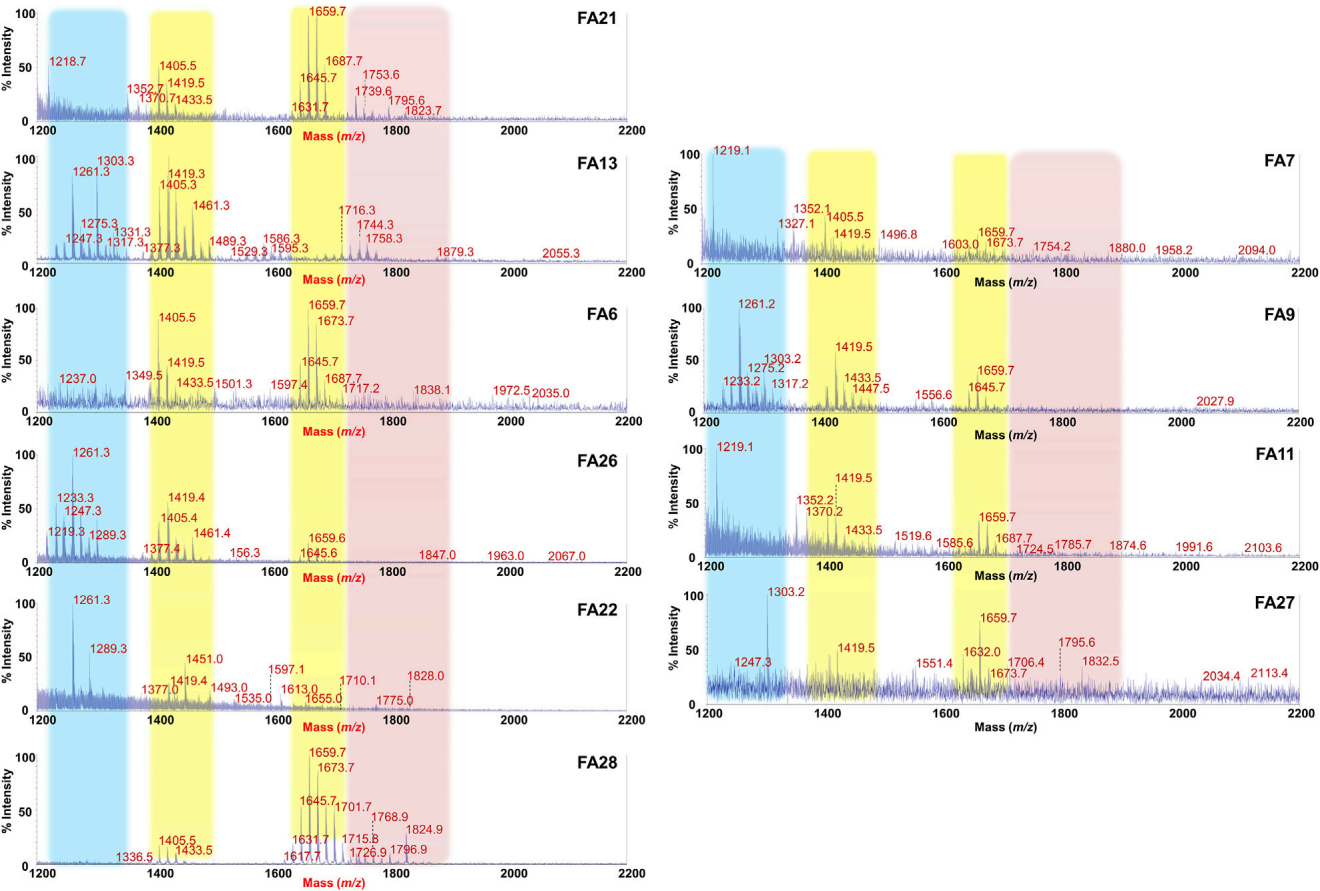
MALDI-TOF mass spectral profile of lipid A mixture extracted from fecal supernatants of food allergy (FA) children. Reflectron MALDI-TOF MS spectra were recorded in negative ion polarity using THAP as the matrix. Yellow boxes highlight the clusters of peaks assigned to *mono*-phosphorylated tetra- (around *m/z* 1,419) and penta-acylated (around *m/z* 1,659) lipid A species from *Bacteroides* sp. Pink box displays families of peaks attributable to potentially pro-inflammatory LPS, i.e., *bis*-phosphorylated and/or hexa-acylated lipid A species. Light blue box highlights the cluster relative to *bis*-phosphorylated tri-acylated lipid A species. Spectra were labeled “FA” followed by a number where FA indicates a food allergic child while the number is unique for each patient and is used to trace the patient information in the collection of samples of the MATFA project.

Lipid A is the primary immunostimulatory part of an LPS, as it is recognized by the immunity receptor complex Toll-like receptor 4 (TLR4)/Myeloid differentiation factor-2 (MD-2), leading to the production of pro-inflammatory mediators that initiate and shape the immune response (Di Lorenzo et al., 2022). However, the activation of the TLR4/MD-2-dependent response is greatly dependent on the chemical structure of the lipid A (Di Lorenzo et al., 2013; Di Lorenzo et al., 2015b; Molinaro et al., 2015; Garcia-Vello et al., 2022b). In order to enable a rapid profiling of lipid A from feces and therefore to take a glance at the fecal LPS immunostimulatory potential, we have conceived an adaptation of a micro-scale lipid A isolation procedure (El Hamidi et al., 2005) in order to extract the lipid A directly from an aliquot of each fecal supernatant. All the fecal lipid A mixtures (CT Lipid A and FA Lipid A) were inspected by negative and positive-ion MALDI-TOF MS and MS/MS. Reflectron MALDI-TOF MS spectra, recorded in negative-ion polarity, of all CT Lipid A and FA Lipid A are reported in Figures 2, 3. Strikingly, MALDI-TOF MS spectra of all CT Lipid A were almost identical, indicating the occurrence of a predominant lipid A type in feces of healthy pediatric donors. Noteworthy, all CT Lipid A had masses with a *m/z* ratio of <1720, which is consistent with the presence of hypo-acylated lipid A species, that is lipid A expressing less than six acyl chains. Moreover, it was possible to promptly associate these peaks to *Bacteroides*-derived lipid A as in all MS spectra (Figure 2) two main clusters of peaks were detected and matched with [M-H]^−^ *mono*-phosphorylated penta-acylated (at about *m/z* 1,659) and tetra-acylated lipid A species (at about *m/z* 1,419), in agreement with previous studies on *Bacteroides* sp. lipid A (Weintraub et al., 1989; Vatanen et al., 2016; Jacobson et al., 2018; Di Lorenzo et al., 2020; Pither et al., 2022b). Moreover, each of these groups of peaks was characterized by the occurrence of mass differences of 14 amu (a–CH_2_– unit) that is diagnostic of the presence of lipid A species differing in the acyl chain length, also in accordance to the well-known length heterogeneity of fatty acids decorating *Bacteroides* sp. lipid A (Weintraub et al., 1989; Vatanen et al., 2016; Jacobson et al., 2018; Di Lorenzo et al., 2020; Pither et al., 2022a). To confirm this structural hypothesis, a detailed negative-ion MS/MS analysis has been conducted on several ion peaks (Supplementary Figure S3) that allowed to determine the location of the phosphate group and the acyl moieties with the respect to the disaccharide backbone. Briefly, the main peak at *m/z* 1,659 observed in most of the MS spectra was assigned to a mixture of at least four isomers all consistent with penta-acylated lipid A species made up of the typical glucosamine disaccharide backbone carrying one phosphate on the reducing glucosamine unit and bearing 17:0(3-OH) and/or 16:0(3-OH), and 17:0(3-OH), 16:0(3-OH) and/or 15:0(3-OH) as primary amide- and ester-bound acyl chains respectively, and one secondary 15:0 unit, with the latter present as acyloxyacylamide of the nonreducing glucosamine. The minor peak at *m/z* 1,419 was assigned to a *mono*-phosphorylated tetra-acylated lipid A species lacking in the primary *O*-linked acyl moiety of the nonreducing glucosamine. Overall MS and MS/MS data of the CT Lipid A were consistent with the isolation and presence of almost exclusively lipid A, and therefore of LPS, belonging to *Bacteroides* sp., which was in agreement with our previous metagenomic analyses (De Filippis et al., 2021).

By contrast, a remarkable difference and a high heterogeneity of components was found in MALDI-TOF MS spectra of FA Lipid A in which the identification of a common trend was not straightforward. As shown in Figure 3, although clusters of peaks attributable to *Bacteroides*-derived lipid A (at about *m/z* 1,659 and *m/z* 1,419) could still be detected in most of the cases, several key differences compared to CT Lipid A spectra were immediately apparent. Briefly, i) the majority of MS spectra displayed a lower signal-to-noise ratios compared to CT counterparts; ii) a significant reduction in the intensity of peaks assigned to *mono*-phosphorylated penta-acylated lipid A species was observed in some spectra together with an increment in the intensity of peaks ascribed to *mono*-phosphorylated tetra-acylated ones; iii) in some spectra, masses with a *m/z* ratio of >1,720 were detected. This latter characteristic was investigated further as it was considered an indication of the presence of *bis*-phosphorylated and/or hexa-acylated lipid A species, and therefore of potentially more pro-inflammatory LPSs. A negative-ion MS/MS analysis has been performed and allowed to establish the structure of several lipid A not detected in CT feces (Supplementary Figure S4). Choosing as examples MS spectra of patients FA21 and FA28, it was possible to identify *bis*-phosphorylated penta-acylated lipid A (at *m/z* 1753.6 and *m/z* 1795.6) (Supplementary Figures S4A,MB), and a Proteobacteria-like *bis*-phosphorylated hexa-acylated lipid A (at *m/z* 1796.9) (Supplementary Figure S4C). Finally, another common trait amongst FA Lipid A MALDI-TOF MS spectra was the occurrence of a cluster of peaks at about *m/z* 1261.3, which was assigned to *bis*-phosphorylated tri-acylated lipid A species devoid of the primary ester bound acyl chains and of the secondary acyl substituent(s) (Supplementary Figure S4D).

### 3.3 GM-derived LPS and lipid A from FA children elicited a Th2 response

**FIGURE 4:**
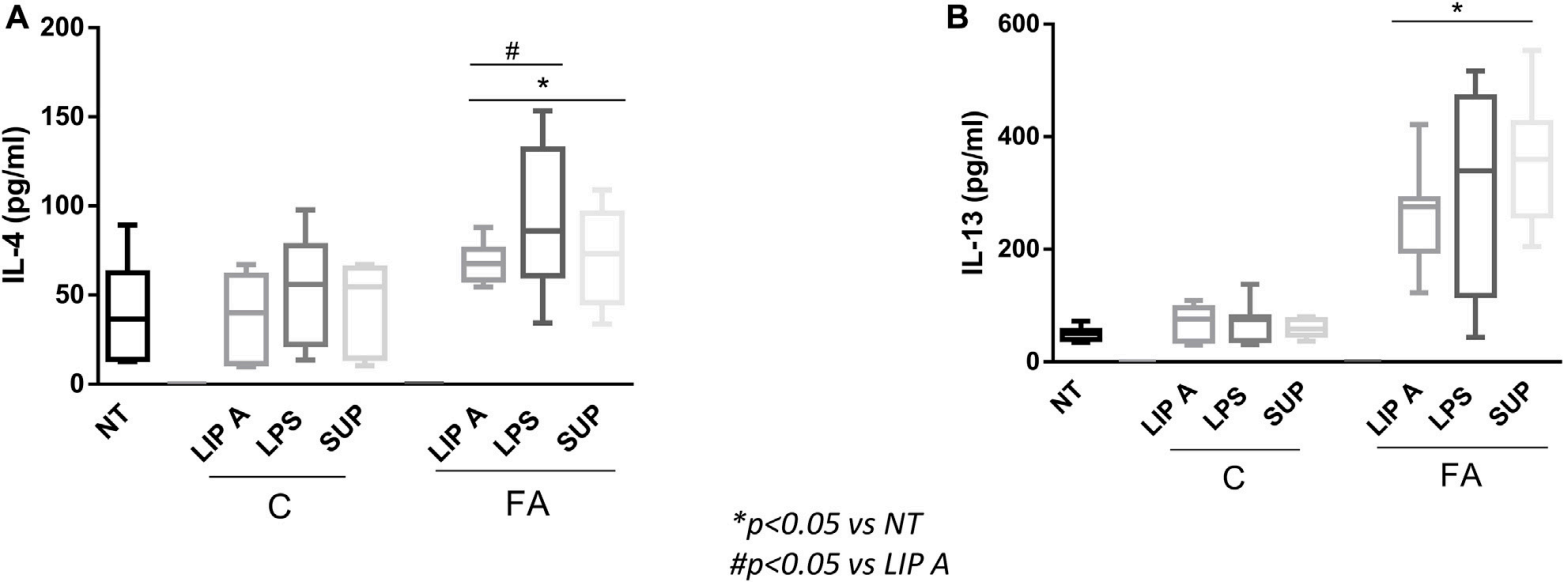
Fecal lipid a (LIP A), LPS and supernatants (SUP) from FA children elicit a pro-allergic Th2 cytokines response in human peripheral blood mononuclear cells (PBMCs). PBMCs from healthy children (*n* = 3) were exposed to 100 μg/mL of fecal lipid A, LPS and supernatants. The stimulation for 24 h with LIP A, LPS and SUP induced a significant increase of IL-4 **(A)** and IL-13 **(B)** production by PBMCs. Each component was tested in duplicate on three different PBMCs samples. Data are expressed as mean ± standard deviation. *p < 0.05 vs NT (not treated) cells; #*p* < 0.05 vs*.* Lip A, as defined by one-way ANOVA test, followed by Tukey post-hoc test. C stands for healthy controls.

The LPS released by gut microbes can act locally and, after crossing the gut barrier and entering circulation, also systemically (Mohr et al., 2022). In the frame of allergic diseases, LPS was shown to either promote or prevent T helper 2 (Th2) cell allergic responses (Eisenbarth et al., 2002; Delayre-Orthez et al., 2004; Kim et al., 2007; Bortolatto et al., 2008), but the underlying mechanism(s) remain unclear. To provide a causal relationship between the presence of a specific LPS/lipid A chemistry and a potential ability in eliciting an allergic response, we tested fecal LPSs from CT and FA children, as well as FA and CT lipid A along with their related fecal supernatants on PBMCs from healthy pediatric donors. We chose PBMCs as they are a valuable tool in food research to perform studies within the FA field (Drago et al., 2015; Lozano-Ojalvo et al., 2015; Paparo et al., 2021) but also because they contain LPS responsive cells similar to those present in the gut, thus representing a common proxy for mucosal leukocytes (Sokol et al., 2008; Ardeshir et al., 2014). Remarkably, only stimulation with lipid A and LPS obtained from FA patients induced an increased production of the Th2 cytokines IL-4 and IL-13 (Figure 4, panels A, B).

### 3.4 GM-derived LPS and lipid A from healthy children activated immune tolerance mechanisms from FA children

**FIGURE 5:**
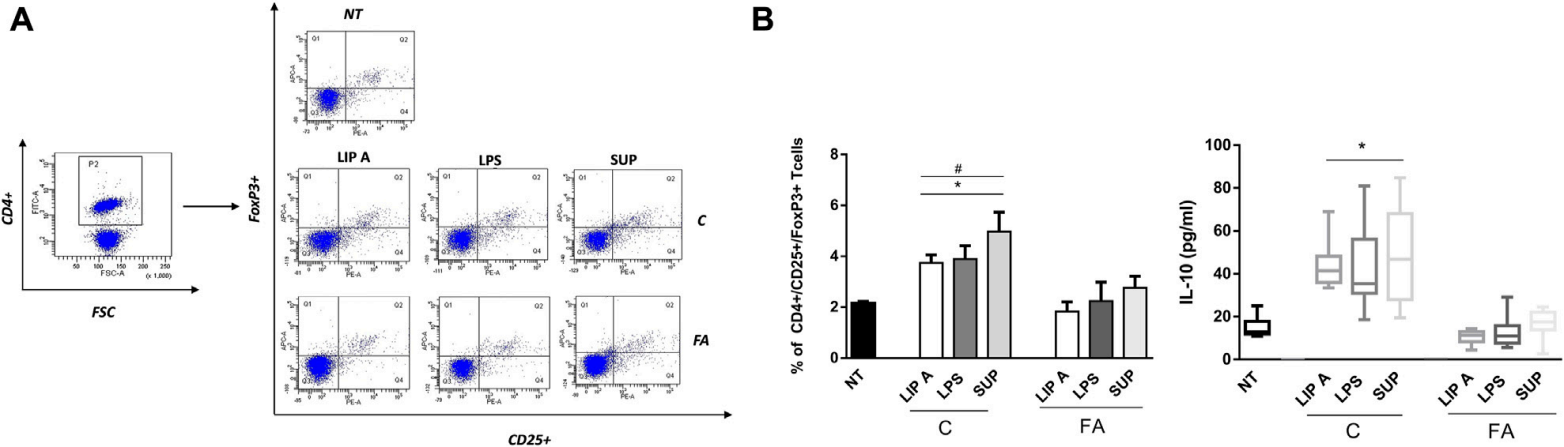
Fecal lipid A (LIP A), LPS and supernatants (SUP) from healthy children (C) induced immune tolerance mechanisms activation. PBMCs from healthy children (*n* = 3) were exposed to 100 μg/mL of fecal lipid A (LIP A), LPS and supernatants for 3 days. Cells were harvested for flow cytometry analysis, and the supernatants were collected for cytokines analysis. **(A)** Representative dot plots obtained by flow-cytometry after staining with CD4^+^/CD25+/FoxP3^+^. The stimulation for 24 h with LIP A, LPS and SUP elicited a significant increase the percentage of CD4^+^/CD25^+^/FoxP3^+^ Tregs. **(B)** This effect paralleled with a significant increase of IL-10 production by PBMCs. Each component was tested in duplicate on three different PBMCs samples. Data are expressed as mean ± standard deviation. *p < 0.05 vs*.* NT (not treated) cells; #*p* < 0.05 vs*.* LIP A, as defined by one-way ANOVA test, followed by Tukey post-hoc test.

To explore the effects of fecal lipid A and LPS on the main drivers of immune tolerance, we investigated regulatory T cells (Tregs) and IL-10 production in PBMCs from healthy children stimulated with lipid A, LPS and fecal supernatants. We observed that lipid A, LPS and fecal supernatants from CT elicited a significant increase of CD4^+^/CD25^+^/FoxP3^+^ cells (Figure 5, panel A). This effect significantly correlated with the production of the tolerogenic cytokine IL-10 (r = 0.9668, *p* < 0.001). This effect paralleled with a significant increase in the production of the tolerogenic cytokine IL-10. No modulation was observed upon stimulation with lipid A, LPS and fecal supernatants from FA children (Figure 5, panel B).

### 3.5 GM-derived LPS from healthy children activated non-classical monocytes and IL-12 production

**FIGURE 6:**
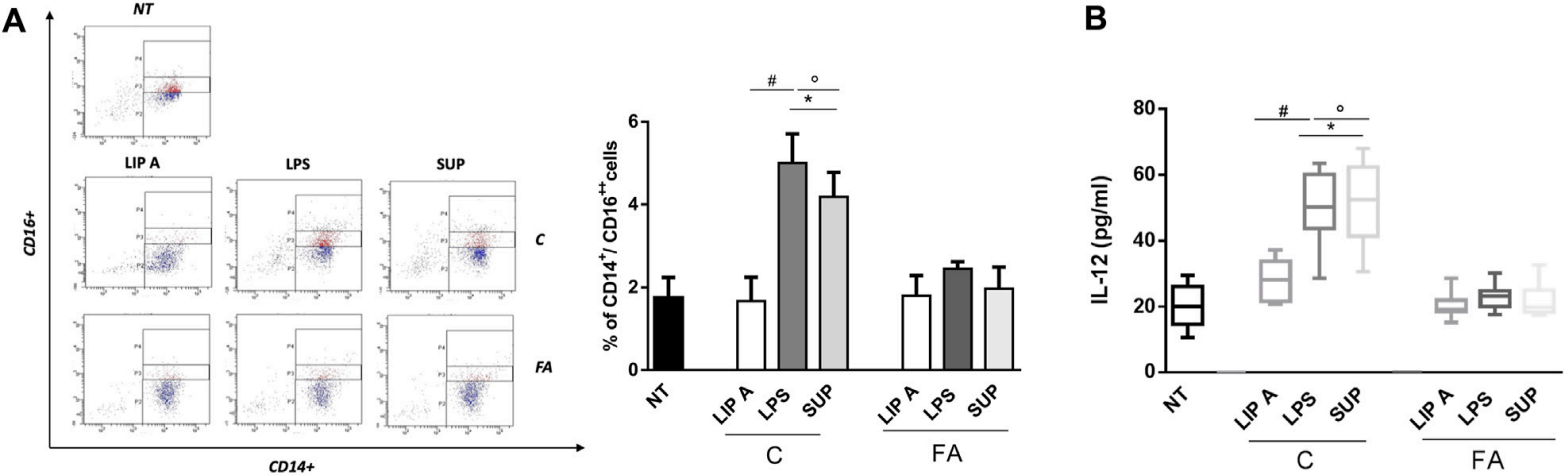
Fecal LPS and supernatant (SUP) from healthy children (C) activated non-classical monocytes and IL-12 production inhibiting Th2-oriented response. PBMCs from healthy children (*n* = 3) were exposed to 100 μg/mL of fecal lipid A (LIP A), LPS and supernatants for 3 days. Cells were harvested for flow cytometry analysis, and the supernatants were collected for cytokines analysis. **(A)** Representative dot plots obtained by flow-cytometry after staining with CD14^+^/CD16^++^. The stimulation for 24 h with LPS and SUP from healthy controls elicited a significant increase of CD14^+^/CD16^++^ cells number. No modulation was observed for stimulation with LIP A. **(B)** IL-12 production by PBMCs resulted in a significant increase after stimulation with LPS and SUP from healthy children. No modulation was observed for stimulation with LIP A. Each component was tested in duplicate on three different PBMCs samples. Data are expressed as mean ± standard deviation. *p < 0.05 vs*.* NT (not treated) cells; #*p* < 0.05 vs*.* LIP A; °*p* < 0.05 vs*.* LPS, as defined by one-way ANOVA test, followed by Tukey post-hoc test.

It has been demonstrated that LPS can promote or suppress Th2 cell sensitization depending on activation of classical and non-classical monocytes (Kaur et al., 2021). These cells control the capacity of dendritic cells to produce IL-12, which in turn led to the suppression of the Th2 cell differentiation program in allergen-specific T cells (Bachus et al., 2019; Bachus et al., 2019). In order to evaluate the potential ability of fecal lipid A and LPS derived from healthy controls in reducing the allergic response, we assessed non-classical monocytes and IL-12 production in PBMCs from healthy children. Notably, the exposure to CT LPS and fecal supernatants significantly increased CD14^+^/CD16^++^ cells number compared to FA lipid A, LPS and fecal supernatants (Figure 6, panel A). Concomitantly, IL-12 release resulted in a significant increase after stimulation with CT LPS and fecal supernatants (Figure 6, panel B).

## 4 Discussion

Pediatric FA is growing at alarming rate with increasing socio-economic burden and negative impact on the quality of life (Spolidoro et al., 2023). Studies have reported that GM could modulate the occurrence of FA (Eisenstein, 2020; Han et al., 2021; Spolidoro et al., 2023), although the bacterial components involved in this phenomenon are still poorly characterized. In this work we attempted to identify the bacterial constituents modulating pro-tolerogenic or pro-allergic mechanisms by focusing on the GM-derived LPS of healthy children or pediatric patients with FA. We found that GM-derived LPSs of FA children chemically differ from LPS collected from healthy controls and that these differences were responsible for divergent activities on human immune cells. In line with our results, previous studies have shown that GM-derived LPSs from healthy adults, predominantly composed of Bacteroidales, were poorly immunostimulatory while exerting an inhibitory action on TLR4 signaling activation (Vatanen et al., 2016; d'Hennezel et al., 2017; Yoshida et al., 2020). Likewise, differences in the dominant GM LPS types and their related immunoactivity were described comparing infants living in rural environments and those living in industrialized countries, with lower levels of *Bacteroides* LPS found in the latter group (Vatanen et al., 2016).

However, in this study we have descended to the molecular scale and reported for the first time the isolation, purification, and chemical characterization of GM-derived LPS in FA and healthy children by providing insights into the carbohydrate and lipid composition as well as in the LPS macromolecular nature. This analysis allowed to identify key chemical and qualitative features distinguishing LPS from FA children vs healthy controls. These differences resulted even more evident when we comparatively analyzed the fecal lipid A mass spectral profile. To the best of our knowledge, this is the first report on chemical/MALDI-TOF MS and MS/MS characterization of GM-derived lipid A executed directly on stool samples, without small-scale (El Hamidi et al., 2005), single-colony (Yang et al., 2022) or large-scale bacterial cultures (Di Lorenzo et al., 2014; Kim et al., 2017), which have been currently representing the conventional methods for isolation/characterization of GM LPS. Our data suggested the presence of a “chemical lipid A barcode” shared among healthy children that is consistent with *mono*-phosphorylated penta- and tetra-acylated lipid A of *Bacteroides* sp. On the contrary, a more heterogenous lipid A mass spectral profile was observed in FA children. The lower signal to noise ratio noticed for FA-associated lipid A MS spectra compared to healthy controls suggested the lack of a predominant Gram-negative species in FA-associated GM and likely, a total minor abundance of Gram-negative species, as also supported by our previous metagenomic analyses (De Filippis et al., 2021) as well as by the low yield of LPS isolated from FA feces in this study (Supplementary Table S2). Nevertheless, thanks to the MS study we were able to identify in FA samples the presence of typical pro-inflammatory lipid A types such as *bis*-phosphorylated and hexa-acylated lipid A species. In addition, it is worth noting that in most MS spectra of stool samples from FA children we observed a significant reduction in the intensity of peaks assigned to *mono*-phosphorylated penta-acylated lipid A species in favor of an increment of the intensity of peaks ascribed to *bis*-phosphorylated tri-acylated and *mono*-phosphorylated tetra-acylated lipid A species (Figure 2). This intriguing feature leads to the hypothesis of a possible altered host processing/inactivation of LPS by host enzymes, such as acyloxyacyl hydrolase (AOAH). AOAH is in charge of removing secondary acyl chains of the LPS and therefore to tremendously decrease their TLR4-immunoactivation potential while they still keep the ability to competitively inhibit TLR4-signalling by active LPS (Munford and Hall, 1986). Acting as such, AOAH is responsible for the maintaining of cell responsiveness to LPS expressed by commensal bacteria thus governing the balance of Th17 and Treg polarization, including host defense and immune-mediated inflammatory diseases (Janelsins et al., 2014). Indeed, Aoah polymorphisms have been correlated with development of rhinosinusitis and asthma (BarnesGrant et al., 2006; Zhang et al., 2012), as well as with intestinal inflammatory conditions (Fairfax et al., 2012). Therefore, our data suggest that not only the occurrence of highly immunostimulatory LPSs may play a role in facilitating allergic response, but also the accumulation of totally inactive LPSs (or even immunoinhibitory LPSs) might represent a distinctive feature contributing to the FA occurrence. These data tend forward the possibility of considering the chemistry of GM-derived LPS as a potential biomarker for diagnosis of pediatric FA, and as a potential target for FA prevention and treatment.

An additional novelty of this study lies in the direct evaluation of the pro-allergic and pro-tolerogenic potential of LPS and lipid A isolated from feces, avoiding the labor-intensive protocols entailing isolation of the bacterial strain(s), their growth *in vitro*, extraction/purification of their LPS/lipid A and, only after these steps, their immunological assay. By harnessing PBMCs from healthy donors, we observed that GM-derived LPS and lipid A from healthy children feces enhanced the release of the tolerogenic cytokine IL-10 and increased the number of activated Treg cells, which in turn are known to play a key role in promoting and maintaining immune tolerance (Sakaguchi et al., 2008; Traxinger et al., 2022). Even more relevant could be the observation that LPSs derived from healthy controls induced the expansion of non-classical monocytes. These monocytes are known to produce granulocyte-macrophage colony-stimulating factor (GM-CSF), whose presence greatly influences the LPS activity switching from pro-allergic to protective (Kaur et al., 2021). Briefly, in the presence of GM-CSF, *de novo* induced monocyte-derived dendritic cells (moDCs) support LPS-driven protection from allergic inflammation by promoting IL-12 production. In contrast, in the absence of GM-CSF, and thus in absence of properly instructed moDCs, LPS exposure enhances Th2 response (Kaur et al., 2021). Our data collectively support the hypothesis that LPS from healthy children is able to inhibit Th2 responses by inducing the differentiation of GM-CSF-producing non-classical monocytes as well as the release of IL-12 but also to maintain tolerance by enhancing the activated Treg cell number and IL-10 production. It is worth noting that the observation that the sole LPS, and not the lipid A, is able to induce the expansion of non-classical monocytes suggests a potential involvement of the LPS carbohydrate portion (core OS and/or O-antigen) in this phenomenon. By contrast, GM-derived LPS and lipid A from FA children elicited a Th2-oriented response through the release of IL-4 and IL-13 that contribute to triggering and maintaining the allergic inflammation (Yu et al., 2016; Tordesillas et al., 2017).

The development of allergen formulations comprising immunomodulatory molecules that act as adjuvants to shift allergen-specific responses from the Th2 to the Th1 phenotype, and to promote tolerogenic Treg responses, is an area of active research (Zubeldia et al., 2019; Feng et al., 2021). In this frame, co-administration or covalent conjugation of allergens with TLR4 modulators is seen as a powerful strategy with promising results (Bortolatto et al., 2008; Mori et al., 2021). Therefore, in this light, we feel that this study could boost the interest in considering GM LPS from healthy subjects, and/or related *ad hoc* synthesized derivatives, as efficient enhancers of the desired outcomes of FA immunotherapy, thus providing alternative routes for current allergen-specific immunotherapy.

The strength of our study is related to the characterization of structural and functional features of GM-derived LPS in a well-defined population of healthy children and of paediatric patients with FA. The main limitations of our study, however, reside in the lack of data on the potential effects of these bacterial molecules on other components of the immune tolerance network, such as enterocytes tight junctions, and mucus layer, as well as in the lack of identification of specific bacterial species and strains responsible for LPS production. Nonetheless, supported by our previous metagenomic data, we could speculate that LPSs of healthy children mostly belong to *Bacteroides vulgatus* and/or *B. dorei* since also our MS data were congruent with literature data (Vatanen et al., 2016; Di Lorenzo et al., 2020; Okahashi et al., 2021). Finally, other limitations derived from the lack of the phenotypic characterization of the Treg population and of the identification of cells responsible for the Th2 cytokines and IL-10 production in PBMCs. In fact, PBMCs contain lymphocytes (T cells, B cells, natural killer cells), monocytes, and dendritic cells, therefore a mixture of signals from the different cells is expected (Kleiveland et al., 2015). In this frame, it has been demonstrated that several cell types in PBMCs are able to produce Th2 cytokines including the CD4^+^ and CD8^+^ T cells (Wills-Karp, 1999; Hinks et al., 2019), basophils (Min et al., 2004; Ma and Gao, 2019), natural killer cells (Yoshimoto and Paul, 1994), mast cells (Plaut et al., 1989), and eosinophils (Dombrowicz and Capron, 2001). In addition, it has also been demonstrated that LPS stimulates Th2 cytokines production from macrophages through MyD88 and TRAM activation (Büttner et al., 1997; Gereda et al., 2000; Mukherjee et al., 2009). The phenotypic characterization of cellular population involved in the observed responses is the object of ongoing and more in-depth studies.

In conclusion, we have highlighted the pro-allergic or pro-tolerogenic potential of GM and assigned it to a specific molecule, the LPS (or better a class of specific LPS molecules) and its chemistry, thus laying the foundations for bridging the gap between correlation and causation in the involvement of specific gut microbial profiles and the development of immune tolerance or FA.

## Data Availability

The original contributions presented in the study are included in the article/Supplementary Material, further inquiries can be directed to the corresponding authors.
