# Supplementary material for: The chemistry of gut microbiome-derived lipopolysaccharides impacts on the occurrence of food allergy in the pediatric age
Source: Front Mol Biosci. 2023 Oct 13;10:1266293. doi: 10.3389/fmolb.2023.1266293 (PMC10606559; doi:10.3389/fmolb.2023.1266293)
Supplement: Supplementary file 1 [file DataSheet1.PDF]

Supplementary Material

The chemistry of gut microbiome-derived lipopolysaccharides impacts on the occurrence of food allergy in the pediatric age

Flaviana Di Lorenzo, Lorella Paparo, Laura Pisapia, Franca Oglio, Molly Dorothy Pither, Roberta Cirella, Rita Nocerino, Laura Carucci, Alba Silipo, Francesca de Filippis, Danilo Ercolini, Antonio Molinaro and Roberto Berni Canani

Supplementary Table 1. Main features of the study population.

Supplementary Table 2. Yield of the LPS extracted from fecal supernatants.

Supplementary Table 3. Monosaccharide composition analysis of fecal LPS.

Supplementary Figure 1. Silver and Coomassie Brilliant Blue staining of SDS-PAGE of fecal LPS.

Supplementary Figure 2. GC-MS chromatogram profiles of fecal LPS fatty acids detected as methyl esters.

Supplementary Figure 3. Negative-ion MALDI MS/MS spectrum of precursor ions at *m/z* 1659.7 and *m/z* 1419.5.

Supplementary Figure 4. Negative-ion MALDI MS/MS spectra of precursor ions at *m/z* 1753.6, *m/z* 1795.6, *m/z* 1796.9, and *m/z* 1261.3.

Supplementary Table 1. Main features of the study population

	Childrens with food allergy	Healthy children
**N.**	10	10
**Male, n (%)**	3 (30)	3 (30)
**Spontaneous delivery, n (%)**	6 (60)	3 (30)
**Born at term, n (%)**	10 (100)	10 (100)
**Birth weight, gr (mean, SD)**	3138 (418)	3151 (445.4)
**Age at enrollment, months (mean, SD)**	56.3 (10.62)	60.7 (8.16)
**Age at food allergy diagnosis, months (mean, SD)**	16.4 (11.84)	-
**Breastfeeding for at least 2 weeks, n (%)**	7 (70)	6 (60)
**Duration of breastfeeding, months (mean, SD)**	11.29 (5.55)	6.83 (2.50)
**Weaning age, months (mean, SD)**	5.3 (0.70)	4.8 (0.64)
**Familial allergy risk, n (%)**	9 (90)	0 (0)

Supplementary Table 2. Yield of the LPS extracted from fecal supernatants of healthy pediatric donors (CT) and food allergic children (FA). The amount of LPS reported was isolated from 300 mg of dried fecal supernatants and are the result of all the purification and "repurification" steps. Data from two independent extractions/purifications are presented as the mean ± SD. CT or FA are followed by a number which is unique for each pediatric patient or donor and is used to trace their information in the collection of samples of the MATFA project.

**Subject**	LPS (mg)	Subject	LPS (mg)
**CT3**	4.15 ± 0.21	FA6	1.6 ± 0.14
**CT7**	3.2 ± 0.01	FA7	1.2 ± 0.14
**CT8**	3.3 ± 0.28	FA9	1.25 ± 0.14
**CT10**	4.2 ± 0.28	FA11	2.05 ± 0.07
**CT12**	3.8 ± 0.71	FA13	1.5 ± 0.01
**CT16**	3.2 ± 0.14	FA21	2.15 ± 0.21
**CT20**	4 ± 0.14	FA22	1.8 ± 0.21
**CT22**	3.55 ± 0.07	FA26	1.6 ± 0.01
**CT25**	3.35 ± 0.21	FA28	2.05 ± 0.07
**CT28**	3 ± 0.14	FA27	1.25 ± 0.07

Supplementary Table 3. Monosaccharide composition analysis of the six **CT** and six **FA** fecal LPS chosen among those that resulted in the highest yield and which were investigated for their immunological properties. The results reported in the table derived by combining of data from compositional analysis, through acetylated methyl glycosides derivatization, and absolute configuration, through acetylated octyl glycosides derivatization.

	**Monosaccharide composition**
**CT3**	L-Rha; L-Fuc; D-Man; D-Gal; D-Glc; D-GalN; D-GlcN; Kdo
**CT10**	L-Rha; L-Fuc; D-Man; D-Gal; D-Glc; D-GalN; D-GlcN; Kdo
**CT12**	L-Rha; L-Fuc; D-Man; D-Gal; D-Glc; D-GalN; D-GlcN; Kdo
**CT25**	L-Rha; L-Fuc; D-Man; D-Gal; D-Glc; D-GalN; D-GlcN; Kdo
**CT20**	L-Rha; L-Fuc; D-Man; D-Gal; D-Glc; D-GalN; D-GlcN; Kdo
**CT22**	L-Rha; L-Fuc; D-Man; D-Gal; D-Glc; D-GalN; D-GlcN; Kdo
**FA6**	L-Rha; L-Ara; D-Xyl; QuiN; D-GalA; D-Man; D-Gal; D-Glc; D-GalN; D-GlcN; L,D-Hep; Kdo
**FA11**	L-Rha; L-Fuc; D-GalA; D-Man; D-Gal; D-Glc; D-GalN; D-GlcN; L,D-Hep; Kdo
**FA21**	L-Rha; L-Fuc; L-Ara; QuiN; L-FucN; D-GalA; D-Man; D-Gal; D-Glc; D-GalN; D-GlcN; L,D-Hep; Kdo
**FA22**	L-Rha; D-Rha; D-Rib; L-FucN; D-Man; D-Gal; D-Glc; D-GlcN; L,D-Hep; Kdo
**FA26**	L-Rha; L-Ara; D-Xyl; D-GalA; D-Man; D-Gal; D-Glc; D-GalN; D-GlcN; L,D-Hep; Kdo
**FA28**	L-Rha; L-Fuc; D-Man; D-Gal; D-Glc; D-GlcN; L,D-Hep; Kdo

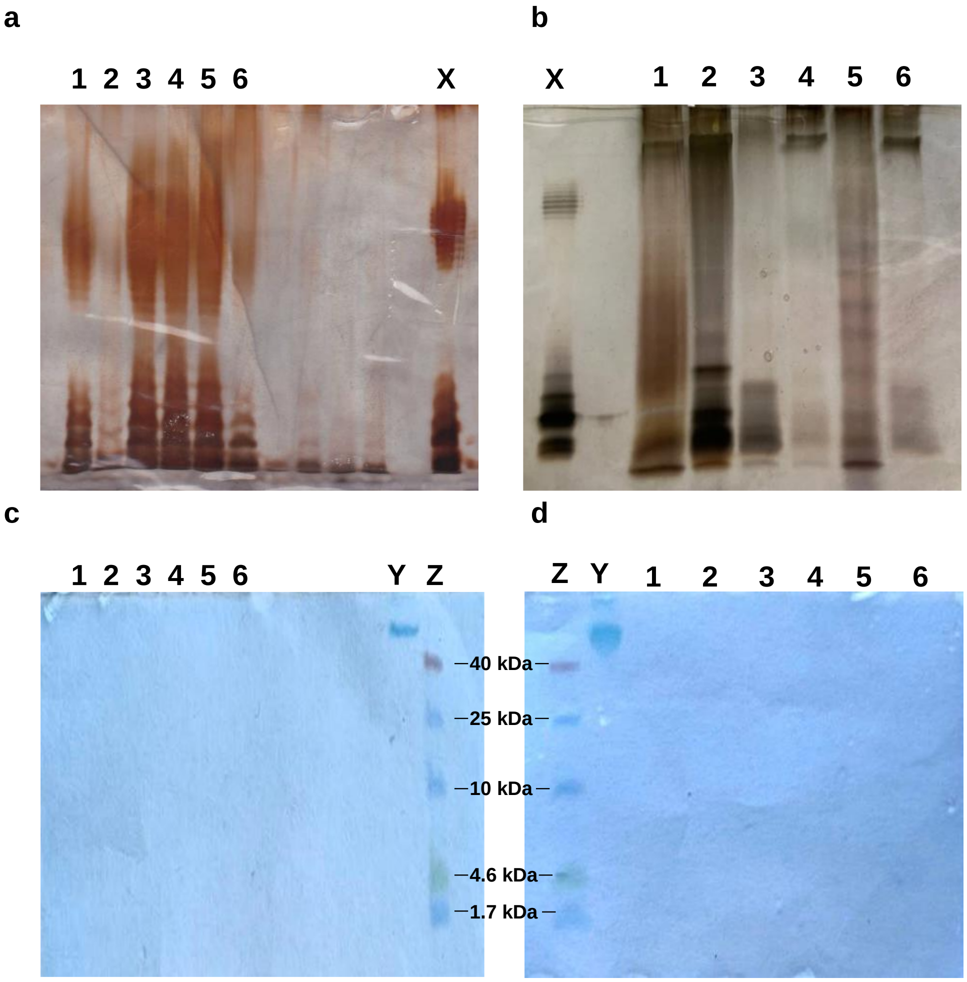

Supplementary Figure 1. Silver (**a**,**b**) and Coomassie Brilliant Blue (**c**,**d**) staining of SDS-PAGE of six LPS extracted from fecal supernatants of CT (**a**,**c**) and FA (**b**,**d**) children chosen as a reference to investigate their immunological properties. In the silver stained SDS-PAGE gel (**a**,**b**) LPS from *E. coli* O127:B8 (8 µL) (Lanes X) was used as a benchmark; 8 µL (Lanes 1-6) of 1 mg/mL solution of each fecal LPS extract were loaded on the gel. In the Coomassie Brilliant Blue stained SDS-PAGE gel (**c**,**d**) bovine serum albumin (BSA) (8 µL) (Lanes Y) and BLUeye Prestained Protein Ladder (2 µL) (Lanes Z) were used as references. Once again 8 µL (Lanes 1-6) of 1 mg/mL solution of each fecal LPS extract were loaded on the gel.

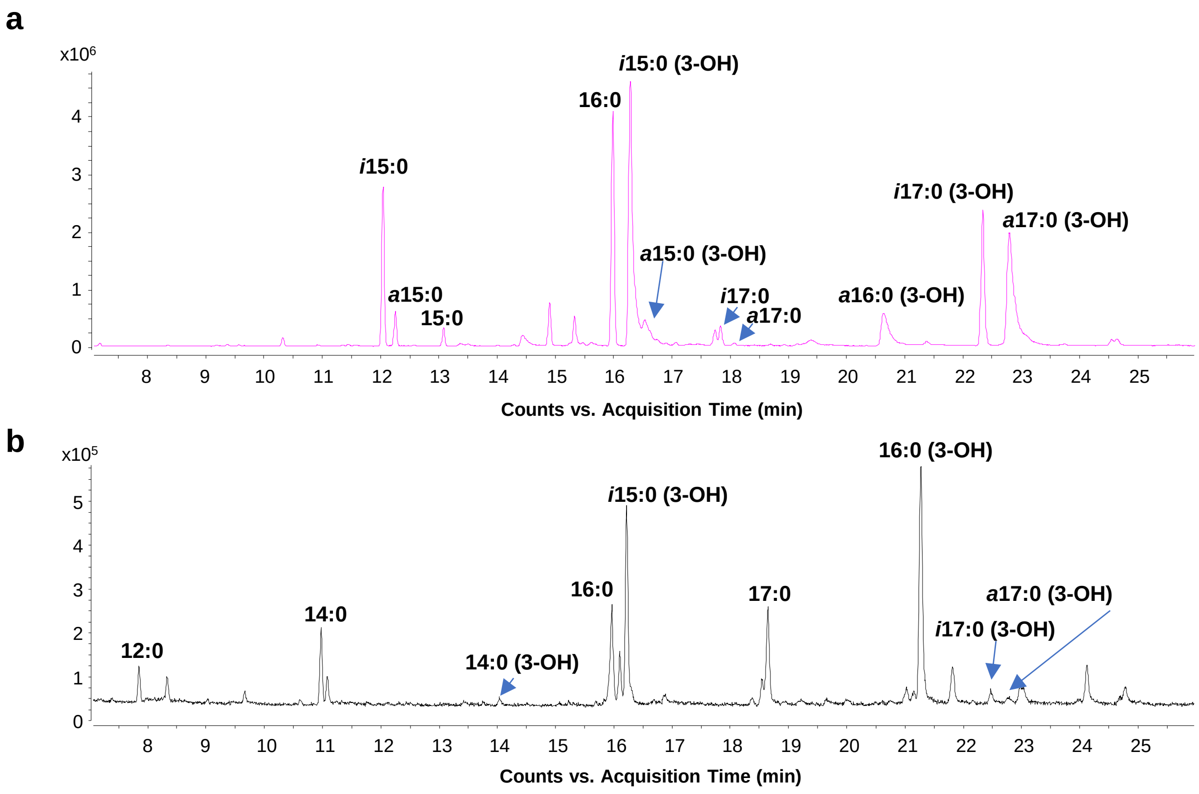

Supplementary Figure 2. Zoom of GC-MS chromatogram profiles recorded after methanolysis followed by hexane extraction of an aliquot of LPS from CT (**a**) and FA feces (**b**) chosen as representatives of the fecal LPS analyzed in this study. The chosen LPS were from CT8 and FA13. By this approach fatty acids are detected as methyl esters and are assigned both by analyzing their fragmentation pattern and by comparison with opportunely prepared fatty acid methyl ester derivatives used as references. The chromatograms highlighted a diverse fatty acid content between CT and FA fecal LPS. "*i*" stands for "*iso*", "*a*" stands for "*anteiso*" and refer to the branched acyl chains.

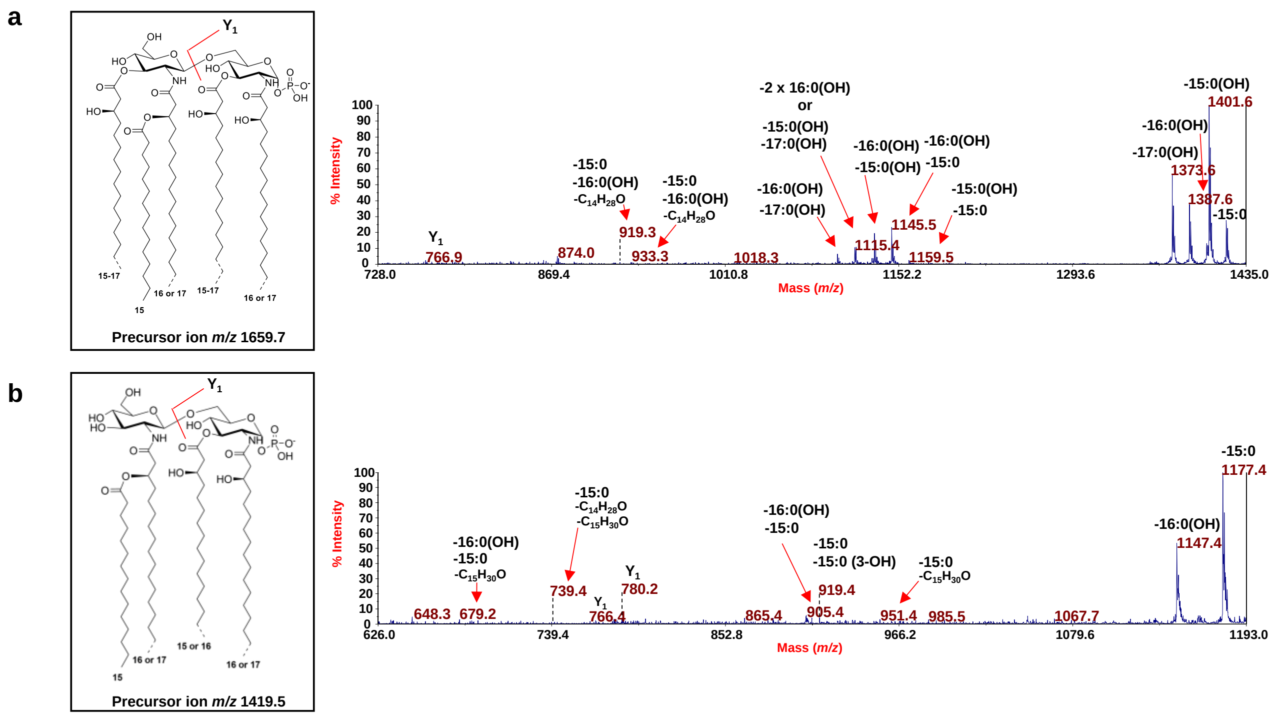

Supplementary Figure 3. Negative-ion MALDI MS/MS spectrum of precursor ions at *m/z* 1659.7 (**a**) and *m/z* 1419.5 (**b**), representative ion peaks of the cluster ascribed to *mono*-phosphorylated penta-acylated and tetra-acylated lipid A species, respectively detected in all CT and in some FA feces. The assignment of main fragments is reported in the spectra alongside with the proposed structure that is sketched in the inset. The observation of the Y-type ion(s), derived by the cleavage of the glycosidic linkage, was key to validate the location of the phosphate group and the nature of the acyl chains decorating the reducing glucosamine. Peaks derived by the loss of $C_{15}H_{30}O$ (226 mass units) or $C_{14}H_{28}O$ (212 mass units), due to the rearrangement occurring on *N*-linked 3-OH acyl chains having the hydroxyl group free, have been also reported. The observation of these fragmentations together with the absence of any peak attributable to a lipid A fragment originating from the loss of a whole unit comprising a hydroxylated acyl chain bearing the secondary 15:0 moiety enabled the positioning of the latter as a substituent of the primary amide-linked fatty acid of the non-reducing glucosamine. Acyl chains in the depicted lipid A structure are given in their unbranched form for description purposes only. Dotted lines indicate the heterogeneity in the length of the fatty acid chains.

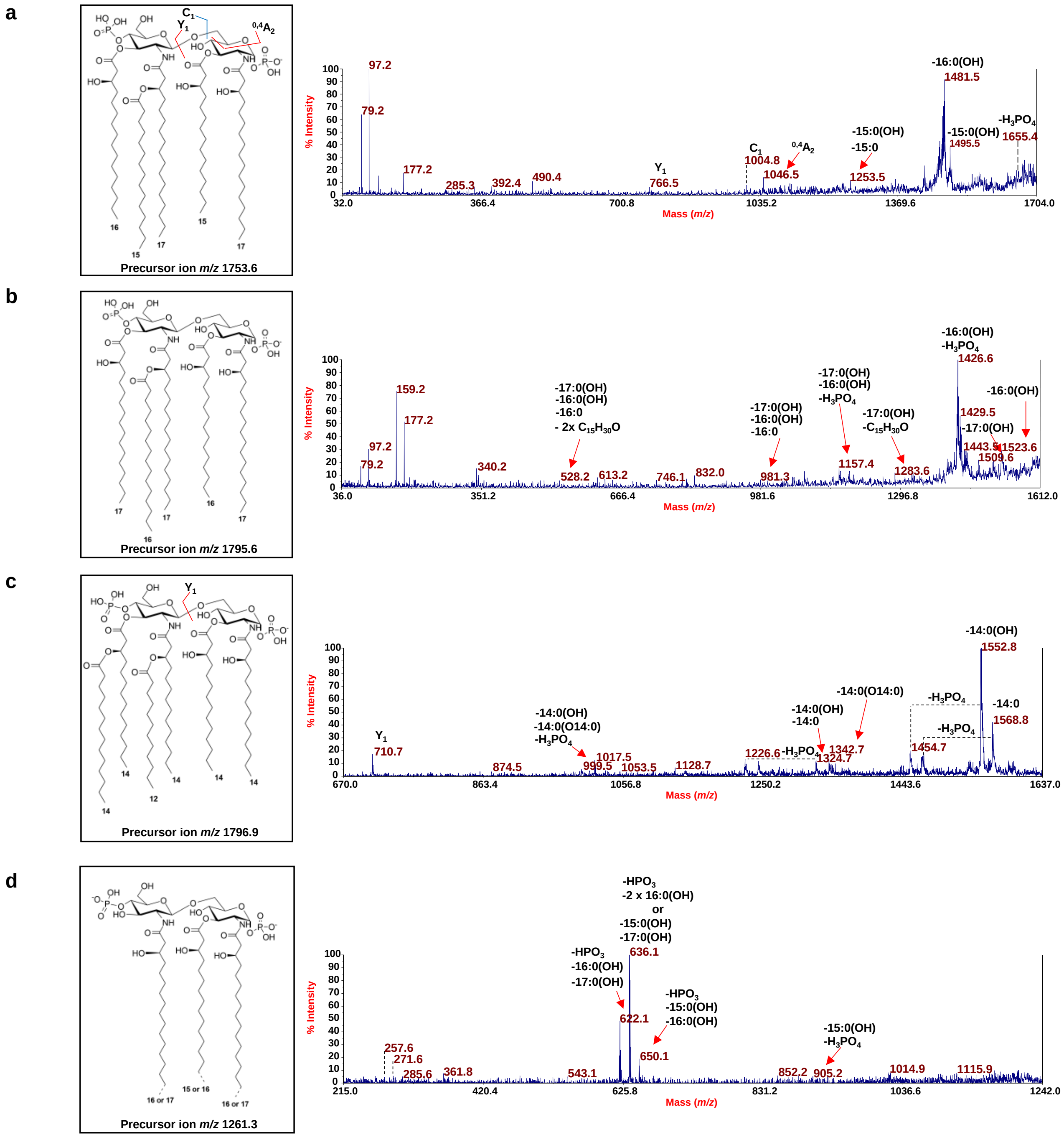

Supplementary Figure 4. Negative-ion MALDI MS/MS spectra of precursor ions at *m/z* 1753.6 (**a**), *m/z* 1795.6 (**b**), and *m/z* 1796.9 (**c**), chosen as representative ion peaks of the cluster ascribed to *bis*-phosphorylated lipid A species found only in FA feces. Negative-ion MALDI MS/MS spectrum of precursor ions at *m/z* 1261.3 (**d**), representative of the family of peaks ascribed to *bis*-phosphorylated tri-acylated lipid A species. The assignment of main fragments is reported in the spectra alongside with the proposed structure that is sketched in the inset. Dotted lines indicate the heterogeneity in the length of the fatty acid chains. Peaks derived by the loss of $C_{15}H_{30}O$ (226 mass units), due to the rearrangement occurring on *N*-linked 3-OH acyl chains having the hydroxyl group free, have been also reported.
